# Exploring the Role of *FICD*, a New Potential Gene Involved in Borderline Intellectual Functioning, Psychological and Metabolic Disorders

**DOI:** 10.3390/genes15121655

**Published:** 2024-12-23

**Authors:** Mirella Vinci, Donatella Greco, Maria Grazia Figura, Simone Treccarichi, Antonino Musumeci, Vittoria Greco, Rossella Pettinato, Angelo Gloria, Carla Papa, Salvatore Saccone, Concetta Federico, Francesco Calì

**Affiliations:** 1 Oasi Research Institute—IRCCS, 94018 Troina, Italy; mvinci@oasi.en.it (M.V.); dgreco@oasi.en.it (D.G.); mfigura@oasi.en.it (M.G.F.); streccarichi@oasi.en.it (S.T.); amusumeci@oasi.en.it (A.M.); vgreco@oasi.en.it (V.G.); rpettinato@oasi.en.it (R.P.); agloria@oasi.en.it (A.G.); cpapa@oasi.en.it (C.P.); 2 Department Biological, Geological and Environmental Sciences, University of Catania, Via Androne 81, 95124 Catania, Italy; salvatore.saccone@unict.it (S.S.); concetta.federico@unict.it (C.F.)

**Keywords:** AMPylation, deAMPylation, adenyltransferase, next-generation sequencing

## Abstract

**Background/Objectives:** AMPylation is a post-translational modification involving the transfer of adenosine monophosphate (AMP) from adenosine triphosphate (ATP) to target proteins, serving as a critical regulatory mechanism in cellular functions. This study aimed to expand the phenotypic spectrum associated with mutations in the FICD gene, which encodes an adenyltransferase enzyme involved in both AMPylation and deAMPylation. **Methods:** A clinical evaluation was conducted on a patient presenting with a complex clinical profile. Whole-exome sequencing (WES) was performed to identify potential genetic variants contributing to the observed phenotype. **Results:** The patient exhibited borderline intellectual functioning (BIF), acanthosis, abdominal muscle hypotonia, anxiety, depression, obesity, and optic nerve subatrophy. WES revealed a de novo missense variant, c.1295C>T p.Ala432Val, in the FICD gene. This variant, classified as of uncertain significance, is located in the highly conserved region TLLFATTEY (aa 428–436), suggesting a potential impact on protein function. **Conclusions:** These findings highlight the importance of the FICD gene in diverse clinical manifestations and emphasize the need for further studies to elucidate the genetic mechanisms underlying these phenotypes. Continued research is essential to improve our understanding of FICD-related conditions.

## 1. Introduction

Protein folding and quality control processes within the endoplasmic reticulum (ER) are vital for maintaining proteostasis and overall cellular health. Disruptions in these processes may lead to the accumulation of misfolded proteins, which is associated with various diseases, including cancer and neurodegenerative disorders [1,2]. To counteract these fluctuations in unfolded proteins, the regulation of chaperone proteins is essential for promoting cellular homeostasis. Within this context, AMPylation helps adjust the activity of chaperones, enabling them to respond effectively to the varying levels of unfolded proteins [3,4]. AMPylation relies on the molecular function of Fic (filamentation induced by cAMP) domains, originally identified in the *E*. *coli* fic-1 gene, which is essential for cAMP-induced filamentation [5]. As widely documented, genes that contain the Fic domain exhibit a high degree of conservation across eukaryotic genomes [6,7,8]. This conservation underscores the evolutionary importance of Fic-domain-containing proteins in various biological processes, particularly in the regulation of protein activity through modifications such as AMPylation [8,9].

Among the various genes associated with the AMPylation enzymatic reaction, FICD is particularly notable due to its essential role. This gene encodes the FIC domain protein adenyltransferase, a bifunctional enzyme capable of both AMPylation and deAMPylation activities. AMPylation is a post-translational modification that involves the transfer of adenosine monophosphate (AMP) from adenosine triphosphate (ATP) to target proteins. Similar to phosphorylation, AMPylation serves as a key regulatory mechanism, modulating the activity of the modified protein. Currently, FICD is recognized as the only enzyme with AMPylation activity in humans [10].

AMPylation is a modification that inactivates proteins, specifically adjusting the activity of BiP, the primary chaperone in the endoplasmic reticulum, to cope with the presence of unfolded proteins. FICD (HYPE), a single ER-localized Fic protein, is responsible for both adding (AMPylation) and removing (deAMPylation) AMP groups from BiP. However, the mechanism that determines when FICD switches between these two activities remains unclear [11]. As previously outlined, the AMPylation activity involving FICD exerts an essential role in neurogenesis processes [12]. The eukaryotic deAMPylation mechanism reveals that the conserved Fic domain residue Glu234 acts as a gatekeeper, inhibiting AMPylation while promoting deAMPylation catalyzed by dimeric FICD. Monomerization increases Glu234 flexibility, switching FICD between its opposing activities, even though both unmodified and AMPylated BiP substrates engage similarly [11]. As documented by various studies and annotated in the OMIM database, *FICD* was associated with Autosomal recessive spastic paraplegia-92 (SPG92) [13] and a MIM phenotype number was already assigned (MIM #620911). Specifically, the previously mentioned study was conducted on consanguineous families, describing the variant inheritance pattern as autosomal recessive (AR). Furthermore, an in vivo Drosophila model of FICD was developed to investigate hereditary spastic paraplegia (HSP). The findings highlighted that loss of Fic function leads to locomotor impairment, reduced levels of the molecular chaperone BIP, and increased reactive oxygen species (ROS) [14]. In addition, it was recently outlined employing murine models that the loss of FicD results in the alteration of unfolded protein response (UPR) signaling and metabolism regulation in the fasting and feeding liver [15].

In the current manuscript, a genetic variant was identified within the *FICD* gene in a subject exhibiting a broad spectrum of symptoms, encompassing acanthosis, abdominal muscle hypotonia, anxiety, depression, obesity, hypotonia, and optic nerve subatrophy. This study aims to explore the potential association between the patient’s symptoms and the *FICD* gene, which has previously been linked to hereditary spastic paraplegia (HSP). By investigating this relationship, we hope to shed light on the broader implications of *FICD* gene mutations and their role in diverse phenotypic presentations.

## 2. Materials and Methods

### 2.1. Libraries Preparation and NGS Analysis

Genomic DNA was obtained from peripheral blood leukocytes of both the patient and the healthy parents, as previously documented [16]. Library preparation (TRIOS) and exome enrichment were performed employing the Agilent SureSelect V7 Kit (Santa Clara, CA, USA), following the manufacturer’s instructions. The sequencing run was conducted utilizing the Illumina HiSeq 3000 instrument (San Diego, CA, USA). This specific method enabled the achievement of 97% of regions, covering at least 20×. The identified variants were filtered based on (i) a recessive/de novo/X-linked pattern of inheritance; (ii) allele frequencies (minor allele frequency, MAF) < 1%, using, as a reference, the following genomic datasets: 1000 Genomes, ESP6500, ExAC, and GnomAD. The reference genome employed for the alignment was HG38. The confirmation of the de novo genetic variant was performed by conventional Sanger sequencing using the BigDyeTM Terminator v1.1 Cycle Sequencing Kit (Life Technologies, Carlsbad, CA, USA) with the SeqStudio Genetic Analyzer instrument (Thermo Fisher Scientific, Waltham, MA, USA). The sequences of the primers adopted for the Sanger experiment were forward: 5′-CGCCAAGTGTACTGAGACCA-3; reverse: 5′-TTGAACCCAGAGTGGTTGGG-3′. Consistently to a previous protocol, DNA fingerprint analysis was conducted to confirm maternity and paternity for both the patient and parents [17].

### 2.2. Data Analysis

The variant was searched on the Human Gene Mutation Database (HGMD Professional 2024) and the Leiden Open Variation Database (LOVD). Furthermore, it was filtered by VarAft (2.17-2) [18]. The identified variant was classified by following the “American College of Medical Genetics” (ACMG) guidelines [19] utilizing the VarSome interface, according to previous research [20]. The prediction of the inheritance pattern of the *FICD* gene was performed by the DOMINO tool (https://domino.iob.ch/) (accessed on 25 September 2024), which assigns the probability for a gene to be dominant according to a probability score ranging from 0 (recessive) to 1 (autosomal dominant) [21].

The gene expression pattern of *FICD* was investigated on Human Protein Atlas (HPA) https://www.proteinatlas.org/) (accessed on 6 June 2024) database. The PhyloP100way score was retrieved from VarSome analysis and was used to analyze the conservation tendency of the specific variant nucleotide region [22]. Gene ontology (GO) terms related to the protein and functional domain annotations were obtained via the QuickGO database (https://www.ebi.ac.uk/QuickGO/) (accessed on 25 September 2024), in addition to the Uniprot (https://www.uniprot.org/) (accessed on 25 September 2024). The IEDB database (https://www.iedb.org/) (accessed on 25 September 2024) was used for search details related to the annotated FICD epitope “TLLFATTEY” (aa 428–436). The specific reaction entry codes involving the FICD enzyme were obtained by Reactome (https://reactome.org) (accessed on 25 September 2024) database.

Protein structure predictions were generated employing the UCSF ChimeraX software version 1.7. The structure analysis was conducted based on the AlphaFold algorithm, generating five models and selecting the “best model”, as previously described [23]. Protein–protein interactions based on experimental studies were investigated on the STRING database. Cytoscape (https://cytoscape.org/) (accessed on 25 September 2024) and Integrated Network and Dynamical Reasoning Assembler (INDRA) GO (https://www.indra.bio/) (accessed on 25 September 2024) tools were used for obtaining the ontology entry the shared pathways between FICD and HSPA5 (BiP) proteins.

## 3. Results

### 3.1. Clinical Report

The patient examined in the present study is a girl, born of non-consanguineous parents. She presents a negative family history of intellectual disabilities. She was born at 39 weeks of gestation via cesarean section due to intrauterine growth restriction and placental aging, with a birth weight of 2.25 kg. The pregnancy was complicated by threatened miscarriage starting from the first trimester, treated pharmacologically. At birth, no asphyxia or cyanosis was reported. At 1.5 months of age, she was hospitalized for an episode of bronchiolitis. Subsequently, recurrent episodes of upper respiratory tract infections, gastroesophageal reflux, aspiration pneumonia, and pericarditis were reported. Psychomotor developmental milestones were achieved with slight delays (first steps at 18 months, sphincter control around 3 years, babbling at 12 months).

The clinical phenotype includes a flat occiput, horizontally oriented palpebral fissures, telecanthus, broad nasal bridge, anteverted nostrils, low-set ears, a flat philtrum, an inverted “V” upper lip, short neck, Dubois’ sign, bilateral webbing of the hands, and flat feet.

At the age of 4 years, a normal female karyotype was noted (46, XX) and an analysis of subtelomeric regions was reported as normal. Investigations including ECG, EMG, ENG, and EEG were normal; brain MRI revealed no evidence of parenchymal damage or malformations. Ventricles and sulci were within normal limits. The midline structures and posterior cranial fossa appeared normal. The optic nerves were moderately reduced in thickness in the intermediate orbital segment; the chiasm was within normal limits. CGH array analysis: Technology: Human Genome CGH 60K Oligo Microarray kit (AMADID 21924, Agilent Technology, Santa Clara, CA, USA), Spatial resolution: Median of 41.5 Kb, Criterion applied: at least four consecutive significantly unbalanced probes, genomic imbalances not reported in the Database of Genomic Variants (http://projects.tcag.ca/variation/index.html) (accessed on 25 September 2024) or in apparently healthy individuals analyzed in the laboratory were noted. The reference DNA was NA15510. No genomic imbalances with a likely pathogenic role were detected.

The patient was previously admitted to our Institute (Associazione IRCCS Oasi Maria SS, Troina, Italy) and discharged with a diagnosis of borderline intellectual functioning (BIF), mixed anxiety–depressive syndrome, subatrophy of the left optic nerve, accentuated lumbar lordosis, abdominal muscle hypotonia, and obesity, and is currently undergoing treatment with paroxetine. Clinical conditions have improved since starting the medication, with reduced irritability and improved mood. However, the patient struggles to get up and sometimes rises as late as 2 p.m. When seated on the couch, she frequently falls asleep. According to her mother, she spends most of her time at home, either in bed or on the couch. The patient also reports irregular menstrual cycles. She weighs 83.7 kg, is 157 cm tall, and has a BMI of 34, with waist and hip circumferences both at 107 cm. Physical examination revealed acanthosis on the neck and underarms, along with striae rubrae on the abdomen. An MRI was performed using T1- and T2-weighted TSE sequences, FLAIR, SWI, and DWI in multiple planes. No significant signal alterations were detected in the cerebral or cerebellar parenchyma, nor in the brainstem. The ventricular system is aligned and not dilated, and no diffusion restriction was observed. The subarachnoid spaces at the base and convexity are within normal limits, with minor signs of chronic sinusitis. Autorefractometry indicated myopic astigmatism in both eyes, with corrected vision measured at 4/10 in the right eye and 2/10 in the left eye. The examined individual is characterized by broad nasal tips with anteverted nostrils, up-slanted palpebral fissures, wide philtrum, strabismus, OS optic subatrophy, dysmorphic ears (small with hypoplastic lobule attached to the face), accentuation of lumbar lordosis, and hypotonia of the abdominal muscles. Cardiac and thoracic examinations were apparently normal. No diabetes was detected. Neurological examination was normal. The anterior eye segment was normal, though the fundus examination revealed myopic choroidosis in both eyes. Primary corneal reflexes appeared centered, motility was normal, and a mild exophoria was noted. In terms of gynecological history, menarche occurred around age 12, but menstrual cycles are irregular (oligomenorrhea). A transabdominal pelvic ultrasound revealed an anteverted uterus of normal size and structure, and both ovaries were of normal size with a multifollicular appearance. Hormonal tests have been recommended, and a gynecological examination noted hypoplasia of the labia minora. The patient currently presents with a diagnosis of borderline intellectual functioning and a mixed anxiety–depressive disorder with phobic features. During the early stages of development, she exhibited mild psychomotor delay, borderline intellectual functioning, and selective mutism. At present, the clinical picture is complex and characterized by mood alterations, relational and behavioral difficulties, and some functional deficits: mood alterations and irritability, sudden shifts from calmness to severe irritability, with intolerance to rules and unavoidable environmental stimuli, leading to episodes of verbal and, less frequently, physical aggression. Relational difficulties such as feelings of inadequacy compared to peers and inability to take initiative due to fear of rejection are noted, as well as the presence of apathy and anhedonia.

### 3.2. NGS Analysis

**Figure 1:**
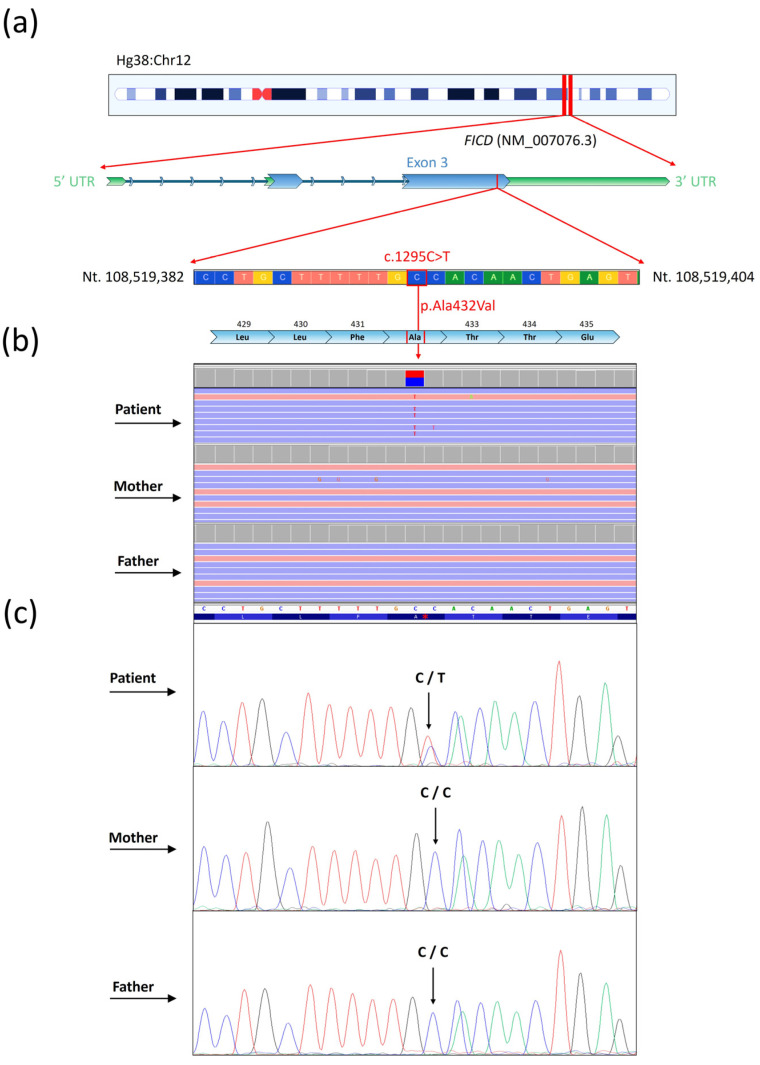
Graphical representation of Next-Generation Sequencing (NGS) analysis for identifying the c.1295C>T variant within the *FICD* gene (NM_007076). (**a**) Chromosomal localization of the *FICD* gene, with a specific focus on the DNA and amino acid sequences surrounding the c.1295C>T (p.Ala432Val) variant. (**b**) Whole-exome sequencing (WES) analysis of the patient, mother, and father, revealing the heterozygous de novo c.1295C>T variant in the patient. This variant is clearly visible using the Integrative Genome Viewer (IGV) tool. (**c**) Confirmation of the variant through conventional Sanger sequencing for the patient, mother, and father.

Before the WES analysis, the patient underwent Array-CGH and MLPA (*PAX7*), both of which yielded normal results. WES analysis did not identify genetic variants in known genes associated with the patient’s phenotype. The analysis unveiled a de novo genetic variant within the *FICD* (NM_007076) gene (Figure 1) in a heterozygous condition.

**Figure 2:**
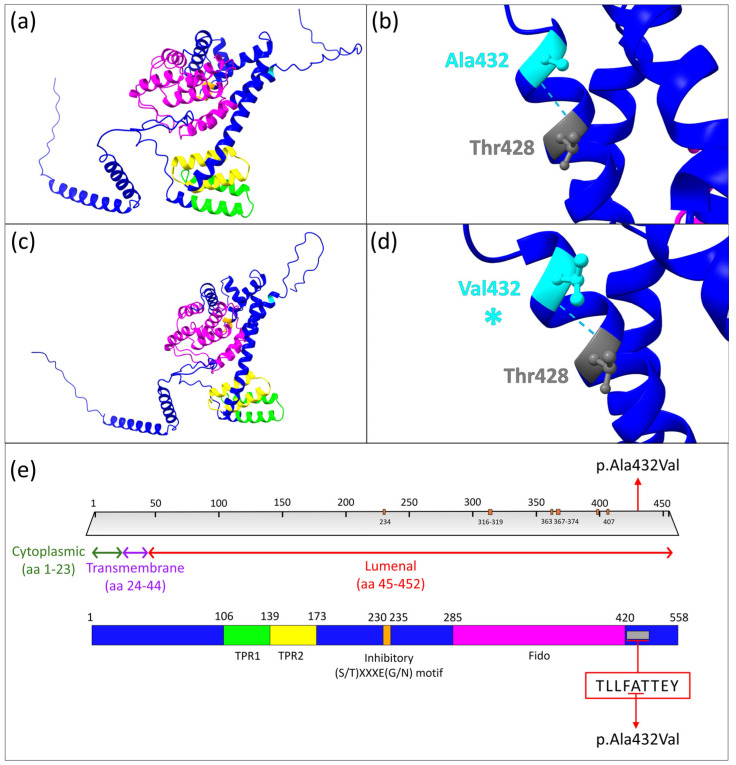
Graphical representation of the wild-type and mutated FICD protein structure predictions. (**a**) Predicted structure of the wild-type FICD protein. (**b**) Close-up view of the wild-type Ala432 residue forming a hydrogen bond with Thr428. (**c**) Predicted structure of the mutated FICD protein. (**d**) Close-up of the mutated Val432 residue establishing a hydrogen bond interaction with Thr428. (**a**–**d**) were generated using the UCSF ChimeraX protein modeling software. (**e**) Graphical representation of the functional domains within the FICD protein, with domain colors consistent with those used in (**a**,**c**). The mutation site within the conserved “TLLFATTEY” (aa 428–436) motif is highlighted by a red arrow. (**e**) was modified from the UniProt database. As annotated by the Uniprot database, FICD has several active and binding sites, precisely located at positions 234, 316–319, 363, 367–374, 399–400, and 407.

As indicated by DOMINO and OMIM databases, the *FICD* gene displays a very likely autosomal recessive inheritance pattern (DOMINO score of 0.132). The variant was localized within the exon 3, at position c.1295C>T. This variant caused the missense mutation p.Ala432Val within the protein FICD. The variant was described as likely pathogenic according to the ACMG criteria while the allele frequency was not found in the GnomAD database. The conservation parameter PhyloP, exhibited a score of 7.905, indicating the high conservation of the specific site of the genetic variant, across the genome of 99 vertebrates. Specifically, the identified mutation was localized within the luminal region (aa 45–458) and in the linear peptidic epitope “TLLFATTEY” (aa 428–436), highly conserved (Figure 2).

### 3.3. Protein Structure Prediction

The structure prediction analysis carried out using the AlphaFold algorithm unveiled notable differences within the FICD protein structure. As predicted, the wild-type protein showed a total number of 392 hydrogen bonds. Conversely, the mutated protein accounted for a total number of 401 hydrogen bonds within the protein structure. It is worth mentioning that there were no predicted differences in the hydrogen bond number and patterns for the mutated residue (Val432) in comparison to the wild type one (Ala432) (as clearly evidenced in Figure 2). In the wild-type FICD protein, several key hydrogen bonds contribute to its structure and function. For instance, Ser8 forms a hydrogen bond with Val9, while Lys16 interacts with Glu14. Leu42 also establishes a bond with Ala39, an interaction unique to the wild-type protein. Additionally, Thr73 interacts with Ala72, and Ser79 forms a bond with Ser77. Lys97 engages with Glu49, and Arg118 forms two hydrogen bonds (with Leu114 and Glu115), while Arg182 forms three bonds with Val178, Asn179, and Glu151. Arg244 participates in two interactions (with Glu221 and Leu240), and Glu259 interacts with Ser257. In the wild-type form, Gln261 establishes two bonds (with Lys256 and Ser257), Asn339 forms three bonds (with Val285, Val335, and Gln336), and Ser340 forms three bonds (with Glu342 and Trp337). In contrast, the mutated FICD protein exhibits different bonding patterns. Notably, Trp21 forms a hydrogen bond with Trp17, which is absent in the wild-type. Leu44 and Glu49 interact with Leu42 and Ala46, respectively, in the mutated form, while Gln50 forms two bonds (with Ala46 and Val47) compared to the single bond with Ala46 in the wild type. The mutated protein shows only one interaction between Cys51 and Val47, whereas the wild type has two. Ser63 uniquely interacts with Leu61 in the mutant, and Leu82 forms a bond with Thr80, which is absent in the wild type. The mutant Arg118 forms an additional bond with Glu115, bringing the total to three bonds (two with Glu115 and one with Leu114). However, mutated Arg182 only forms one bond with Val178. Arg244 in the mutant version forms four bonds (three with Glu221 and one with Leu240), compared to only two in the wild type. Gln261 in the mutated form shows an additional bond with Val253, Lys256, and Ser257. Mutated Asn339 forms four hydrogen bonds (with Val285, Val335, and Gln336), while Ser340 forms only two bonds with Trp337. Lastly, Arg371 in the mutant forms a bond with Asp367, and both Glu435 and Asn446 interact with Phe431 and Gln444, respectively, which are interactions absent in the wild type. As indicated by the DOMINO and OMIM databases, the *FICD* gene displays a very likely autosomal recessive inheritance pattern (DOMINO score of 0.132). The variant was localized within exon 3, at position c.1295C>T.

### 3.4. Protein–Protein Interactions

**Figure 3:**
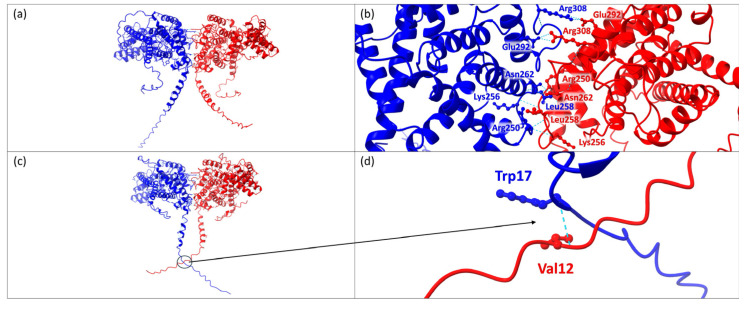
Protein–Protein Interaction and Hydrogen Bond Variations in FICD Homodimerization. (**a**) Graphical representation of the wild-type FICD homodimer, with one FICD protein shown in blue. (**b**) Close-up of hydrogen bonds within the wild-type FICD homodimer. Blue labels mark residues from one FICD molecule, while red labels indicate interacting residues from the second molecule. (**c**) Graphical representation of the FICD homodimer involving the p.Ala432Val mutant (blue) and the corresponding partner FICD (red). A black circle and arrow highlight the additional hydrogen bond between Trp17 of the mutant and Val12 of the partner protein. (**d**) Detailed view of the Trp17-Val12 interaction unique to the mutated FICD homodimer.

Protein–protein interaction analysis using the AlphaFold algorithm predicts that the wild-type FICD forms multiple hydrogen bonds during homodimerization. Key interactions include Ala252 and Lys256 (with bond distances of 3.060 Å and 3.061 Å), Leu258 and Arg250 (2.931 Å and 2.940 Å), and Asn262 with itself (2.904 Å and 2.907 Å) (Figure 3).

Additionally, each FICD in the homodimer forms two hydrogen bonds between Arg308 and Glu292, with bond distances of 3.118 Å, 3.215 Å, 3.085 Å, and 3.189 Å. The interaction between FICD carrying the p.Ala432Val mutation and wild-type FICD exhibits the same hydrogen bond pattern as the wild-type homodimer. The only difference is an additional hydrogen bond predicted between Trp17 of the mutated protein and Val12 of the corresponding FICD dimer, with a bond distance of 2.602 Å.

**Figure 4:**
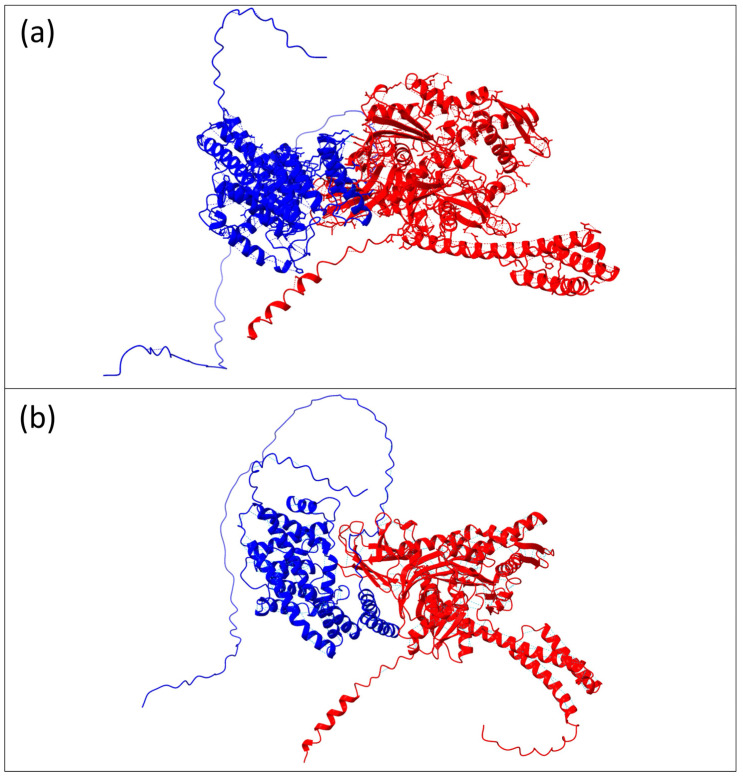
Graphical representation of the predicted interaction between FICD and HSPA5 (BiP). (**a**) Representation of the protein–protein interaction involving the wild-type FICD (blue color) and HSPA5 (red color) proteins. (**b**) Prediction of the interaction between the FICD protein (blue color) carrying the mutation p.Ala432Val and HSPA5 (red color).

In addition, the protein–protein structure prediction was also conducted to assess the variation between the interaction involving both the wild-type and mutated FICD protein and BiP. In particular, the analysis reveals both conserved and distinct hydrogen bond interactions between BiP and the wild-type or mutated FICD proteins, highlighting potential structural and functional implications (Figure 4).

Key hydrogen bonds, such as those involving Thr80 and Thr462, Ser83 and Ser452, and Asn111 and Asp413, are consistently observed in both wild-type and mutant forms, with slight variations in bond distances (Table 1 and Table 2).

However, unique interactions emerged in the mutated FICD, including a novel bond between Ser79 and Ser455 (2.791 Å) and a more robust interaction between Gln496 of BiP and Ala232 of the mutant FICD (3.409 Å), replacing the wild-type interaction with Ile233. Notably, bonds present in the wild type, such as Arg396 and Gln496 (2.562 Å), were absent in the mutant, while the Lys521–Gly317 interaction disappeared entirely.

## 4. Discussion

The current study aimed to expand the phenotypic spectrum associated with the *FICD* gene, which has previously been linked to hereditary spastic paraplegia (HSP) with the MIM phenotype number 620911. The subject examined in this study presents a clinical profile that also includes borderline intellectual functioning (BIF), obesity, abdominal muscle hypotonia, anxiety, depression, acanthosis, and optic nerve subatrophy. In this context, NGS analysis did not identify mutations in known genes related to the patient’s phenotype. The known genes associated with the patient’s phenotype (as defined by the HPO terms) have been listed in the Supplementary Files. It is worth noting that no potential causative variants were identified within this set of genes as a result of the WES data analysis. However, we cannot rule out the possibility that genetic variants in intronic regions not covered by WES, or epigenetic mechanisms, may contribute to the patient’s complex phenotype. Given its complexity, a multifactorial origin of the condition cannot be excluded. Chromosomal anomalies, such as duplications and deletions, have been ruled out, as Array-CGH analysis displayed normal results. Additionally, MLPA analysis of the *PAX7* gene, which encodes a transcription factor involved in muscle stem cell proliferation and myogenesis and is associated with skeletal muscle disorders, was also performed [24]. The result of the previously mentioned MLPA analysis was normal. The identified variant is a de novo mutation, described as uncertain significance, with no reported frequency in the GnomAD database. Moreover, the variant is located in a highly conserved region (PhyloP = 7.905). Furthermore, the mutation described is located within the epitope “TLLFATTEY”, as documented in the IEDB database (epitope ID 1590047) (amino acids 423–436). The FICD gene is annotated in the OMIM database for spastic paraplegia 92 (MIM phenotype entry code #620911), with an autosomal recessive (AR) inheritance pattern. Nevertheless, to date, according to HGMD and LOVD databases, only a few variants have been described as causative. Furthermore, according to the ClinVar database, no pathogenic variants have been detected. Our WES analysis did not identify any additional mutations, including those with a minor allele frequency (MAF) > 1%, within the coding regions or splice sites analyzed. However, while unlikely, it remains plausible that a variant located in a region not covered by WES could contribute to the presumed compound heterozygosity. Given the clinical presentation described in this study, we propose the possibility of an autosomal dominant inheritance pattern for the *FICD* gene associated with the phenotypic condition of the individual examined in this study. Furthermore, we cannot draw definitive conclusions regarding the gene’s expressivity, as heterozygous variants in *FICD* have been previously reported without any established clinical manifestations. We speculate that the mutation identified in the current study alters the interaction between FICD and BiP. Notably, the mutation identified in this study is located in a different position than the previously reported mutations, suggesting that an unknown molecular mechanism could be involved.

In a previous study involving three consanguineous families, the homozygous c.1121G>A (Arg374His) variant within the FICD gene was identified in four individuals [13]. It is worth mentioning that the residue Arg374 was described as a hot spot for variants. The individuals described in the previously mentioned study exhibited a broad spectrum of symptoms similar to those reported in the case presented in this study. Table 3 provides a comprehensive comparison of the phenotype described in this study with those of other individuals carrying variants in the *FICD* gene, either in homozygous or compound heterozygous conditions.

However, unlike the previously documented individuals, our subject has not yet developed motor peripheral neuropathy. Nonetheless, we cannot rule out the possibility of a late-onset manifestation of this condition. Notably, the individual examined experienced mild motor delays in early childhood, taking her first steps at 18 months, although she currently shows no difficulties with walking.

As documented by the HPA database, FICD shows a ubiquitarian expression pattern, involving several human tissues. The gene has been associated with a broad and heterogeneous spectrum of phenotypes. In addition to hereditary spastic paraplegia (HSP) and motor neuron deficits [13,14], the gene has been linked to photoreception defects [26], mild cognitive impairments, including deficits in verbal and nonverbal memory, visuomotor coordination, and processing speed [13], infancy-onset diabetes [13,25], as well as alterations in fasting and feeding liver metabolism [15]. Moreover, alterations in FICD enzymatic activity were implicated in a slow progressive motor neuron disease characterized by spasticity and muscle weakness, primarily affecting the lower limbs. This condition results in walking difficulties, including toe-walking, frequent falls, and an unsteady gait [14]. It is important to note that hereditary spastic paraplegia (HSP) encompasses a group of rare genetic disorders characterized by progressive spasticity and weakness in the lower limbs due to corticospinal tract degeneration [27]. Typically, HSP has an adult onset, though in rare cases, it can manifest during childhood [28]. Common neurological comorbidities in HSP include developmental delay, autism, epilepsy, and attention-deficit/hyperactivity disorder [29]. As previously described, the patient examined in this study currently does not exhibit any motor problems, but these might develop with a late onset. The patient also exhibits metabolic disturbances, as evidenced by a high BMI of 34, indicative of a predisposition to obesity. Moreover, recent studies using murine models have demonstrated that the loss of FicD leads to disruptions in unfolded protein response (UPR) signaling and metabolic regulation in both fasting and feeding states in the liver [15]. In this context, it is well established that dysregulation of UPR signaling can result in significant imbalances in fatty acid metabolism [30]. As previously noted, reversible BiP AMPylation plays a crucial role in protecting the Drosophila visual system under constant light-induced stress. Fic mutant flies exhibit synaptic dysfunction and rhabdomere disintegration after prolonged light exposure, with even heterozygous fic30C null mutants showing heightened susceptibility, similar to homozygous mutants [31]. In this context, the patient in our study presents with optic nerve subatrophy. Interestingly, in a fic heterozygous background, eye-specific expression of the constitutively active AMPylating mutant FicE247G results only in a mildly rough eye phenotype, underscoring the importance of the FICD gene in photoreception [31].

As extensively documented, the AMPylation activity carried out by FICD plays an essential role in neurogenesis [12]. The role of AMPylation in neurodevelopment was evaluated by manipulating the expression of the AMPylating enzyme FICD in cerebral organoids (COs). Downregulation maintained cells in a proliferative progenitor state, while overexpression promoted neurogenesis, suggesting AMPylation as a novel post-translational modification regulating neurogenesis [32].

According to QuickGO annotations, FICD is involved in several molecular functions, including protein binding (GO:0005515), ATP binding (GO:0005524), nucleotidyltransferase activity (GO:0016779), hydrolase activity (GO:0016787), Hsp70 protein binding (GO:0030544), identical protein binding (GO:0042802), protein homodimerization activity (GO:0042803), protein adenylylhydrolase activity (GO:0044603), protein-folding chaperone binding (GO:0051087), and AMPylase activity (GO:0070733). Additionally, FICD plays a role in key biological processes such as the response to unfolded protein (GO:0006986), protein adenylylation (GO:0018117), negative regulation of GTPase activity (GO:0034260), response to endoplasmic reticulum stress (GO:0034976), protein deadenylylation (GO:0044602), and regulation of the IRE1-mediated unfolded protein response (GO:1903894). The enzyme is primarily located in the endoplasmic reticulum (GO:0005783), the endoplasmic reticulum membrane (GO:0005789), and general membranes (GO:0016020).

The Fido domain, known to mediate adenylyltransferase activity [33], displays several structural differences in the FICD protein (residues 285–420) due to the mutation, as predicted by in silico protein structure analysis. In the wild type, Asn339 forms three hydrogen bonds (with Val285, Val335, and Gln336), while Ser340 establishes three bonds (with Glu342 and Trp337), and Arg371 does not interact with Asp367. Ala432, a key residue, forms a hydrogen bond with Thr428. In the mutated FICD protein, Asn339 gains an additional hydrogen bond, bringing the total to four, while Ser340 shows a reduction to two bonds (both with Trp337). The mutated Arg371 forms a new bond with Asp367, absent in the wild type, and Val432 (mutated from Ala432) retains the same interaction with Thr428. Despite these changes, no difference in the hydrogen bond pattern for the mutated Val432 compared to wild-type Ala432 was predicted, yet overall, the mutated FICD protein shows an increase in hydrogen bonds within the Fido domain.

According to the Rhea Database, FICD is classified as an adenylyltransferase enzyme (EC 2.7.7.108). Notably, alterations in adenylyltransferase activity have often been linked to neurological disorders, as this enzyme plays a critical role in axonal protection and maintaining neuronal structural integrity [34,35]. In this context, FICD has been documented to catalyze the reactions annotated as Rhea 55932 and Rhea 54292. As is well known, the dual enzymatic activity of Fic is partially regulated by its dimerization, with the loss of this dimerization leading to increased AMPylation and reduced deAMPylation of BiP [26]. Therefore, the dimerization process appears to play a crucial role in modulating BiP deAMPylation. We propose that the alteration we identified, potentially in conjunction with an additional undetected mutation, may impair the dimerization process, thereby affecting the enzyme’s overall activity. Within this context, the structural prediction carried out using the AlphaFold algorithm displayed an additional hydrogen bond predicted between Trp17 of the mutated protein and Val12 of the corresponding FICD dimer, with a bond distance of 2.602 Å (Figure 3).

As previously highlighted and supported by multiple studies and the STRING database, FICD exhibits a strong protein–protein interaction with Endoplasmic Reticulum Chaperone HSPA5 (also known as BiP). The HSPA5 gene encodes Binding Immunoglobulin Protein (BiP), a member of the Hsp70 family of chaperones localized in the ER lumen. As a highly conserved molecular chaperone, BiP plays a crucial role in various protein folding processes through its two functional domains: the nucleotide-binding domain (NBD) (aa 125–280) and the substrate-binding domain (aa 420–500) (SBD) (Wang et al., 2017). In line with data from Cytoscape and INDRA GO, FICD and HSPA5 are both involved in key pathways such as regulation of IRE1-mediated unfolded protein response (GO:1903894), IRE1-mediated unfolded protein response (GO:0036498), regulation of endoplasmic reticulum unfolded protein response (GO:1900101), endoplasmic reticulum unfolded protein response (GO:0030968), regulation of response to endoplasmic reticulum stress (GO:1905897), cellular response to unfolded protein (GO:0034620), cellular response to topologically incorrect protein (GO:0035967), and response to unfolded protein (GO:0006986). Given this context, we propose that structural variations in the FICD enzyme may impair both the formation of the homodimer and its interaction with HSPA5, potentially disrupting these critical pathways. In fact, the structure prediction analysis related to the interaction between FICD and BiP revealed some important differences. In the mutated FICD, unique interactions emerge, including a novel hydrogen bond between Ser79 and Ser455 (2.791 Å). Notably, some interactions present in the wild type are lost in the mutant, such as the bond between Arg396 and Gln496 (2.562 Å). Additionally, the Lys521-Gly317 interaction is entirely absent in the mutant form.

Functional studies in in vivo models are essential to validate the pathogenicity of the variant in both homozygous and heterozygous states. Furthermore, additional research is crucial to better understand the role of FICD in psychiatric and neuropathic disorders, particularly in light of its potential involvement in metabolic regulation and photoreception.

## 5. Conclusions

FICD is a crucial gene that plays an essential role in AMPylation and deAMPylation processes. Whole-exome sequencing (WES) identified the variant c.1295C>T (p.Ala432Val) in the *FICD* gene in a patient presenting with borderline intellectual functioning, acanthosis, depression, obesity tendencies (BMI 34), and optic nerve subatrophy. This variant, classified as of uncertain significance, has no reported allele frequency in the gnomAD database. While FICD is listed in OMIM as associated with spastic paraplegia and follows an autosomal recessive inheritance pattern, we report a de novo variant in a complex phenotype. We hypothesize the possibility of an autosomal dominant inheritance pattern for the *FICD* gene associated with the phenotypic condition of the individual examined in this study. Structural predictions of the mutated FICD protein involved in homodimerization and interaction with HSPA5 reveal significant differences compared to the wild type, suggesting potential functional implications. This case study strengthens the potential link between FICD and the complex clinical presentation described here. We hope this work will inspire further functional studies to better elucidate the role of *FICD* in human diseases.

## Figures and Tables

**Table 1:** The hydrogen bond pattern that resulted from the in silico interaction between the wild-type FICD and HSPA5 (BiP).

FICD wt Residue	BiP Residue	Distance (Å)
Thr80	Thr462	2.962
Ser83	Ser452	3.017
Thr90	Ser448	2.104
Thr90	Ser448	3.000
Asn111	Asp413	3.185
Asn111	Asp413	2.706
Gln112	Asn239	2.910
Gln112	Asp413	2.879
Lys121	Asp238	2.902
Lys121	Asp238	2.149
Lys124	Asp238	2.654
Lys127	Glu243	2.989
Thr237	Arg492	3.553
Gly317	Glu514	2.734
Arg396	Gln496	2.562
**BiP Residue**	**FICD wt Residue**	**Distance (Å)**
Arg197	Glu105	2.360
Ser448	Thr90	2.077
Gln449	Thr85	3.418
Ser452	Ser83	3.111
Gln458	Glu81	3.399
Thr462	Thr80	2.498
Gln496	Ile233	2.290
Lys521	Gly317	1.993

**Table 2:** The hydrogen bond pattern that resulted from the in silico interaction between the FICD protein carrying the mutation p.Ala432Val and HSPA5 (BiP).

FICD mut Residue	Bip Residue	Distance (Å)
Ser79	Ser455	2.791
Thr80	Thr462	3.078
Ser83	Ser452	3.035
Thr90	Ser448	3.070
Asn111	Asp413	3.435
Asn111	Asp413	2.735
Gln112	Asn239	2.880
Gln112	Asp413	3.124
Lys121	Asp238	2.960
Lys121	Asp238	2.181
Lys124	Asp238	2.680
Lys127	Glu243	3.023
Thr237	Arg492	2.614
Gly317	Glu514	2.924
**Bip Residue**	**FICD mut Residue**	**Distance (Å)**
Gly430	Glu81	3.382
Gln449	Thr85	3.415
Ser452	Ser83	3.182
Ser455	Ser79	2.791
Gln458	Glu81	3.567
Thr462	Thr80	2.641
Gln496	Ala232	3.409
Gln496	Ile233	1.841

**Table 3:** Comparison of the phenotype described in this study carrying the variant c.1295C>T in the heterozygous condition with those of other individuals previously described.

Symptom	c.1295C>T (het)	c.1113G>C (hom) [25]	c.1121G>A (hom) [13]	c.1109_1110delGG; c.1121G>A (comp. het) [13]
Cohort	1	5	4	1
Sex	F ^a^	F, F, F, F, M ^b^	M, M, M, M	F
Walking issues	Slight psychomotor delay: first steps at 18 months	Not detected	Yes	Wheelchair at age 20
Hypotonia	Abdominal muscle	Not described	Not described	Not described
Obesity	Yes	Yes	Not described	Not described
Diabetes	Not detected	Yes	Not described	Not described
Cognitive impairment	Borderline intellectual functioning	Severe	1 none, 3 mild	None
Behavioral and psychiatric symptoms	Mixed anxiety-depressive disorder with phobic features	Not described	2 none, 2 depression	None
Optical issues	Optic nerve subatrophy	3	Not detected	Not detected
Neuropathy	Not detected	Not detected	Mild	Not detected

^a^ F indicates a “female” individual; ^b^ M indicates a “male” individual.

## Data Availability

The data presented in this study are in the main text. Further data are available on request from the corresponding author.

## Supplementary Materials

The following supporting information can be downloaded at: https://www.mdpi.com/article/10.3390/genes15121655/s1, Supplementary Material: List of the known genes associated with patient phenotypes, according to the HPO terms.

## References

[1] Araki K., Nagata K. (2011). Protein Folding and Quality Control in the ER. Cold Spring Harb. Perspect. Biol..

[2] Braakman I., Hebert D.N. (2013). Protein Folding in the Endoplasmic Reticulum. Cold Spring Harb. Perspect. Biol..

[3] Truttmann M.C., Pincus D., Ploegh H.L. (2018). Chaperone AMPylation Modulates Aggregation and Toxicity of Neurodegenerative Disease-Associated Polypeptides. Proc. Natl. Acad. Sci. USA.

[4] Perera L.A., Ron D. (2023). AMPylation and Endoplasmic Reticulum Protein Folding Homeostasis. Cold Spring Harb. Perspect. Biol..

[5] Utsumi R., Kusafuka S., Nakayama T., Tanaka K., Takayanagi Y., Takahashi H., Noda M., Kawamukai M. (1993). Stationary Phase-Specific Expression of the *Fic* Gene in *Escherichia coli* K_-12_ Is Controlled by the *RpoS* Gene Product (*σ^38^*). FEMS Microbiol. Lett..

[6] Kinch L.N., Yarbrough M.L., Orth K., Grishin N.V. (2009). Fido, a Novel AMPylation Domain Common to Fic, Doc, and AvrB. PLoS ONE.

[7] Garcia-Pino A., Zenkin N., Loris R. (2014). The Many Faces of Fic: Structural and Functional Aspects of Fic Enzymes. Trends Biochem. Sci..

[8] Rosani U., De Felice S., Frizzo R., Kawato S., Wegner K.M. (2024). FicD Genes in Invertebrates: A Tale of Transposons, Pathogenic and Integrated Viruses. Gene.

[9] Roy C.R., Cherfils J. (2015). Structure and Function of Fic Proteins. Nat. Rev. Microbiol..

[10] Fauser J., Gulen B., Pogenberg V., Pett C., Pourjafar-Dehkordi D., Krisp C., Höpfner D., König G., Schlüter H., Feige M.J. (2021). Specificity of AMPylation of the Human Chaperone BiP Is Mediated by TPR Motifs of FICD. Nat. Commun..

[11] Perera L.A., Rato C., Yan Y., Neidhardt L., McLaughlin S.H., Read R.J., Preissler S., Ron D. (2019). An Oligomeric State-dependent Switch in the ER Enzyme FICD Regulates AMPYlation and deAMPYlation of BiP. EMBO J..

[12] Kielkowski P., Buchsbaum I.Y., Kirsch V.C., Bach N.C., Drukker M., Cappello S., Sieber S.A. (2020). FICD Activity and AMPylation Remodelling Modulate Human Neurogenesis. Nat. Commun..

[13] Rebelo A.P., Ruiz A., Dohrn M.F., Wayand M., Farooq A., Danzi M.C., Beijer D., Aaron B., Vandrovcova J., Houlden H. (2022). BiP Inactivation Due to Loss of the DeAMPylation Function of FICD Causes a Motor Neuron Disease. Genet. Med..

[14] Lobato A.G., Ortiz-Vega N., Canic T., Tao X., Bucan N., Ruan K., Rebelo A.P., Schule R., Zuchner S., Syed S. (2024). Loss of Fic Causes Progressive Neurodegeneration in a Drosophila Model of Hereditary Spastic Paraplegia. Biochim. Biophys. Acta (BBA)—Mol. Basis Dis..

[15] Casey A.K., Stewart N.M., Zaidi N., Gray H.F., Cox A., Fields H.A., Orth K. (2024). FicD Regulates Adaptation to the Unfolded Protein Response in the Murine Liver. Biochimie.

[16] Calì F., Vinci M., Treccarichi S., Papa C., Gloria A., Musumeci A., Federico C., Vitello G.A., Nicotera A.G., Di Rosa G. (2024). PLEKHG1: New Potential Candidate Gene for Periventricular White Matter Abnormalities. Genes.

[17] Calì F., Forster P., Kersting C., Mirisola M.G., D’Anna R., De Leo G., Romano V. (2002). DXYS156: A Multi-Purpose Short Tandem Repeat Locus for Determination of Sex, Paternal and Maternal Geographic Origins and DNA Fingerprinting. Int. J. Legal. Med..

[18] Desvignes J.-P., Bartoli M., Delague V., Krahn M., Miltgen M., Béroud C., Salgado D. (2018). VarAFT: A Variant Annotation and Filtration System for Human next Generation Sequencing Data. Nucleic. Acids Res..

[19] Richards S., Aziz N., Bale S., Bick D., Das S., Gastier-Foster J., Grody W.W., Hegde M., Lyon E., Spector E. (2015). Standards and Guidelines for the Interpretation of Sequence Variants: A Joint Consensus Recommendation of the American College of Medical Genetics and Genomics and the Association for Molecular Pathology. Genet. Med..

[20] Kopanos C., Tsiolkas V., Kouris A., Chapple C.E., Albarca Aguilera M., Meyer R., Massouras A. (2019). VarSome: The Human Genomic Variant Search Engine. Bioinformatics.

[21] Quinodoz M., Royer-Bertrand B., Cisarova K., Di Gioia S.A., Superti-Furga A., Rivolta C. (2017). DOMINO: Using Machine Learning to Predict Genes Associated with Dominant Disorders. Am. J. Hum. Genet..

[22] Capriotti E., Fariselli P. (2017). PhD-SNPg: A Webserver and Lightweight Tool for Scoring Single Nucleotide Variants. Nucleic. Acids Res..

[23] Vinci M., Greco D., Treccarichi S., Chiavetta V., Figura M.G., Musumeci A., Greco V., Federico C., Calì F., Saccone S. (2024). Bioinformatic Evaluation of KLF13 Genetic Variant: Implications for Neurodevelopmental and Psychiatric Symptoms. Genes.

[24] Costamagna D., Casters V., Beltrà M., Sampaolesi M., Van Campenhout A., Ortibus E., Desloovere K., Duelen R. (2022). Autologous IPSC-Derived Human Neuromuscular Junction to Model the Pathophysiology of Hereditary Spastic Paraplegia. Cells.

[25] Perera L.A., Hattersley A.T., Harding H.P., Wakeling M.N., Flanagan S.E., Mohsina I., Raza J., Gardham A., Ron D., De Franco E. (2023). Infancy-onset Diabetes Caused by De-regulated AMPylation of the Human Endoplasmic Reticulum Chaperone BiP. EMBO Mol. Med..

[26] Casey A.K., Moehlman A.T., Zhang J., Servage K.A., Krämer H., Orth K. (2017). Fic-Mediated DeAMPylation Is Not Dependent on Homodimerization and Rescues Toxic AMPylation in Flies. J. Biol. Chem..

[27] Salinas S., Proukakis C., Crosby A., Warner T.T. (2008). Hereditary Spastic Paraplegia: Clinical Features and Pathogenetic Mechanisms. Lancet Neurol..

[28] Ebrahimi-Fakhari D., Saffari A., Pearl P.L. (2022). Childhood-Onset Hereditary Spastic Paraplegia and Its Treatable Mimics. Mol. Genet. Metab..

[29] Panwala T.F., Garcia-Santibanez R., Vizcarra J.A., Garcia A.G., Verma S. (2022). Childhood-Onset Hereditary Spastic Paraplegia (HSP): A Case Series and Review of Literature. Pediatr. Neurol..

[30] Ward C.P., Peng L., Yuen S., Chang M., Karapetyan R., Nyangau E., Mohammed H., Palacios H., Ziari N., Joe L.K. (2022). ER Unfolded Protein Response in Liver In Vivo Is Characterized by Reduced, Not Increased, De Novo Lipogenesis and Cholesterol Synthesis Rates with Uptake of Fatty Acids from Adipose Tissue: Integrated Gene Expression, Translation Rates and Metabolic Fluxes. Int. J. Mol. Sci..

[31] Moehlman A.T., Casey A.K., Servage K., Orth K., Krämer H. (2018). Adaptation to Constant Light Requires Fic-Mediated AMPylation of BiP to Protect against Reversible Photoreceptor Degeneration. eLife.

[32] Buchsbaum I.Y. (2019). Discovering Novel Mechanisms of Human Cortical Development & Disease Using In Vivo Mouse Model and In Vitro Human-Derived Cerebral Organoids. Ph.D. Thesis.

[33] Worby C.A., Mattoo S., Kruger R.P., Corbeil L.B., Koller A., Mendez J.C., Zekarias B., Lazar C., Dixon J.E. (2009). The Fic Domain: Regulation of Cell Signaling by Adenylylation. Mol. Cell.

[34] Sasaki Y., Vohra B.P.S., Lund F.E., Milbrandt J. (2009). Nicotinamide Mononucleotide Adenylyl Transferase-Mediated Axonal Protection Requires Enzymatic Activity but Not Increased Levels of Neuronal Nicotinamide Adenine Dinucleotide. J. Neurosci..

[35] Pottorf T., Mann A., Fross S., Mansel C., Vohra B.P.S. (2018). Nicotinamide Mononucleotide Adenylyltransferase 2 Maintains Neuronal Structural Integrity through the Maintenance of Golgi Structure. Neurochem. Int..

